# Frequency of ABO and Rh Blood Groups Among Blood Donors in the Hail Region of Saudi Arabia

**DOI:** 10.7759/cureus.69195

**Published:** 2024-09-11

**Authors:** Reem Eltayeb

**Affiliations:** 1 Department of Medical Laboratory Science, College of Applied Medical Science, University of Hail, Hail, SAU

**Keywords:** abo, blood group, blood phenotype, rhesus d, saudi arabia

## Abstract

Objective: Proper blood bank inventory management and safe, efficient blood transfusion services require a thorough understanding of ABO and Rh(D) blood group distributions in specific populations. The objective of this research was to evaluate the distribution of ABO and Rh blood types among different ethnic blood donors in the Hail region of Saudi Arabia and compare the results to those of other populations.

Methodology: Data from 3,166 blood donors were analyzed retrospectively. Blood bank records provided sociodemographic information as well as blood group phenotypes. Descriptive statistics were employed. The distribution of ABO and Rh blood types was statistically examined using the chi-square test.

Results: The study identified a total of 3,166 blood donors, with the majority being males (3,083 (97.4%)). The median age of the donors was 35 years, with an interquartile range of 30-41 years. Of the cohort, 1,425 (45%) consisted of young donors, defined as individuals aged 30-41 years. The O blood type accounted for 1,409 (44.5%) of all types, making it the most prevalent. The next most common blood types were B at 837 (26.4%), A at 741 (23.4%), and AB at 179 (5.7%). When combined, O positive was the most prevalent type, accounting for 1,226 (38.7%) of the total. This was followed by type B positive at 745 (23.5%), type A positive at 651 (20.6%), O negative at 183 (5.8%), AB positive at 171 (5.3%), B negative at 92 (2.9%), A negative at 90 (2.8%), and AB negative at eight (0.3%).

Conclusion: According to the results, out of all the ABO phenotypes, O was the most common. B, A, and AB came next. In addition, Rh(D) positivity was observed in 88.2% of the donors. These results have considerable consequences for blood transfusion strategies in the Hail region.

## Introduction

Blood group systems are complicated genetic determinants of erythroid antigens [1]. The International Society of Blood Transfusion now recognizes 45 such systems, which include around 362 antigens [2]. The ABO and Rh systems are key components of clinical transfusion treatment [3]. The ABO blood types were discovered by Karl Landsteiner, which made modern methods of blood transfusion possible [4]. Landsteiner and Weiner later described the Rh system [5]. The antigens, predominantly glycoproteins or glycolipids, are embedded within the membranes of red blood cells. The *ABO* gene is located on chromosome 9, while the *Rh(D)* gene is situated on chromosome 1 [6,7].

The ABO system is categorized into four distinct phenotypes (A, B, AB, and O), each characterized by a unique combination of antigens and antibodies, based on the agglutination patterns of their red blood cells with the corresponding antibodies. ABO antigens are fully developed after birth and persist throughout an individual's lifetime [8]. On the other hand, the Rh system, specifically the D antigen, is characterized by a greater degree of structural complexity. This system is encoded by two genes, *RHD* and *RHCE*, resulting in an extremely varied system [9].

ABO and Rh antigens are immunogenic; hence, pre-transfusion compatibility testing is required [10]. Numerous studies have investigated the link between blood type antigens and a variety of disorders [11,12]. Geographical and ethnic factors also play a substantial role in the phenotypic distribution of ABO and Rh [13-15].

In Saudi Arabia, O is the most common blood group, followed by A, B, and AB. A significant proportion of the Saudi population is Rh(D) negative [16-20]. Proper blood bank inventory management and safe, efficient blood transfusion services require a thorough understanding of ABO and Rh(D) blood group distributions in specific populations [15,21,22].

Previous studies in the Hail region of Saudi Arabia have recorded the frequency of ABO and Rh, but the sample sizes were small [16]. Additionally, the observed distribution of blood groups in the Hail region has shown variations when compared to findings from other regions in Saudi Arabia, suggesting potential regional differences. This study aimed to expand on these findings by assessing a larger group of blood donors from the Hail region to comprehensively characterize the distribution of ABO and Rh blood types and compare the results to those of other populations.

## Materials and methods

Study design

A retrospective cross-sectional investigation was carried out in the Hail region of Saudi Arabia using the blood bank of a maternity and children's hospital. The frequencies of the ABO and Rh blood types in those admitted between January 1, 2020, and December 31, 2023, were recorded.

Study population

We studied the data of 3,166 blood donors. The ABO and Rh blood group phenotypes, as well as the demographic information of the patients (age, gender, origin, and place of residence), were extracted from both electronic and paper-based blood bank files.

Blood donor inclusion criteria

We used the data of blood donors aged 18-65 years who had hemoglobin levels of 12 g/dL or higher (women) or 13 g/dL or higher (men). The volunteers had to weigh at least 45 kg (350 mL) for women or 50 kg (450 mL) for men and test negative for sexually transmitted diseases (HIV, syphilis, hepatitis B, and hepatitis C). Normal health indicators (temperature and blood pressure) were considered.

Data collection

A uniform document was used to collect data pulled from the blood bank records. Age, gender, nationality, residency, and ABO and Rh blood groups were all noted. The efficacy of blood group reagents was verified by analyzing previously identified blood samples.

Statistical analysis

Data were imported into EpiData version 4.6 (EpiData Association, Odense, Denmark) and analyzed using SPSS version 25 (IBM SPSS Statistics, Armonk, NY). Descriptive statistics were computed. The chi-square test was employed to compare ABO and Rh blood group patterns according to age, gender, nationality, and place of residence. Statistical significance was determined as p < 0.05 at 95% confidence interval.

## Results

The study population comprised a total of 3,166 participants with a median age of 35 years (interquartile range: 30-41 years) and an age range of 18-65 years. Males constituted the greatest proportion of participants, accounting for 97.4% of the total. The 30-39-year-old age group accounted for 45.1% of the total population, which was 3,083 individuals. Of the sample, 77.6% of the population lived in urban areas (2,458 individuals). The percentage of Saudi nationals was 93% (2,943 individuals) (Table 1).

**Table 1:** Frequency and distribution pattern of ABO blood group phenotypes (N=3,166)

Variable		ABO phenotype (number (%))				Test statistics		
		A	B	AB	O	Chi-square	Degree of freedom	P-value
Age years	18-24	78 (10.5)	87 (10.4)	25 (14)	150 (10.6)	17.78	15	0.27
	25-29	90 (12.1)	98 (11.7)	15 (8.4)	173 (12.3)			
	30-34	171 (23.1)	186 (22.2)	34 (19)	342 (24.3)			
	35-39	155 (20.9)	201 (24)	48 (26.8)	288 (20.4)			
	40-44	129 (17.4)	115 (13.7)	27 (15.1)	236 (16.7)			
	45-65	118 (15.9)	150 (17.9)	30 (16.8)	220 (15.6)			
Sex	Male	731 (98.7)	809 (96.7)	174 (97.2)	1,369 (97.2)	6.70	3	0.27
	Female	10 (1.3)	28 (3.3)	5 (2.8)	40 (2.8)			
Residence	Urban	583 (78.7)	636 (76)	130 (72.6)	1,109 (78.7)	5.30	3	0.15
	Rural	158 (21.3)	201 (24)	49 (27.4)	300 (21.3)			
Nationality	Saudi	685 (92.4)	768 (91.8)	169 (94.4)	1,321 (93.8)	4.10	3	0.25
	Non-Saudi	56 (7.6)	69 (8.2)	10 (5.6)	88 (6.2)			

There was no significant difference in the distribution of ABO blood group phenotypes by sex (chi-square: 6.70, degree of freedom: 3, p = 0.08), nationality (chi-square: 4.10, degree of freedom: 3, p = 0.25), or residency. The distribution of Rh(D) phenotypes was not found to be linked with sex (chi-square: 0.92, degree of freedom: 1, p = 0.34), nationality (chi-square: 0.65, degree of freedom: 1, p = 0.42), or residency (chi-square: 1.30, degree of freedom: 1, p = 0.26) (Table 2). However, a statistically significant correlation was found between the Rh(D) phenotype and age (chi-square: 15.58, degrees of freedom: 5, p = 0.008) (Table 2).

**Table 2:** Frequency and distribution pattern of Rh blood group phenotypes (N=3,166)

Variable		Rhesus status (number (%))		Frequency and percentage (number (%))	Test statistics		
		Positive	Negative		Chi-square	Degree of freedom	P-value
Age years	18-24	300 (10.7)	40 (10.7)	340 (10.7)	15.58	5	0.008
	25-29	322 (11.5)	54 (14.5)	376 (11.9)			
	30-34	643 (23)	90 (24.1)	733 (23.2)			
	35-39	636 (22.8)	56 (15)	692 (21.9)			
	40-44	433 (15.5)	74 (19.8)	507 (16)			
	45-65	459 (16.4)	59 (15.8)	518 (16.4)			
Sex	Male	2,717 (97.3)	366 (98.1)	3,083 (97.4)	0.92	1	0.34
	Female	76 (2.7)	7 (1.9)	83 (2.6)			
Residence	Urban	2,177 (77.9)	281 (75.3)	2,458 (77.6)	1.30	1	0.26
	Rural	616 (22.1)	92 (24.7)	708 (22.4)			
Nationality	Saudi	2,600 (93.1)	343 (92)	2,943 (93)	0.65	1	0.42
	Non-Saudi	193 (6.9)	30 (8)	223 (7)			

The pattern of blood groups revealed that the most common was type O, representing 1,409 (44.5%) of donors. With 837 (26.4%) of the population belonging to blood group B, A came in at 741 (23.4%) and AB at 179 (5.7%). A positive Rh(D) blood type was found in 2,793 (88.2%) subjects, whereas a negative Rh(D) type was found in 373 (11.8%). The O positive blood group combination was the most common, making up 1,226 (38.7%) of all cases. This was followed by B positive at 745 (23.5%), A positive at 651 (20.6%), O negative at 183 (5.8%), AB positive at 171 (5.3%), B negative at 92 (2.9%), A negative at 90 (2.8%), and AB negative at eight (0.3%) (Table 3).

**Table 3:** Combined distribution of ABO and Rh blood groups in Hail region

Blood groups		Male			Female				Total		
		Number		%	Number	%			Number	%	
A Rh negative	88			2.9%	2		2.4%	90			2.8%
A Rh positive	643			20.9%	8		9.6%	651			20.6%
B Rh negative	91			3%	1		1.2%	92			2.9%
B Rh positive	718			23.3%	27		32.5%	745			23.5%
AB Rh negative	7			0.2%	1		1.2%	8			0.3%
AB Rh positive	167			5.4%	4		4.8%	171			5.3%
O Rh negative	180			5.8%	3		3.6%	183			5.8%
O Rh positive	1,189			38.6%	37		44.6%	1,226			38.7%
Total		3,083	100%		83		100%	3,166			100%

## Discussion

The pattern of blood groups revealed that the most common, representing 44.5% of donors, was the O blood type. With 26.4% of the population belonging to blood group B, A came in at 23.4%, and AB at 5.7%. A positive Rh(D) blood type was found in 88.2% of the subjects, whereas a negative Rh(D) type was found in 11.8%. The O positive blood group was the most common, accounting for 38.7% of all cases.

Approximately two-thirds (67.7%) of the donors were below the age of 40 years, which is consistent with similar observations made in Ethiopia [23], Uganda [24], Tanzania [25], and Saudi Arabia [26]. Younger persons frequently have higher donation rates because of their superior physical health, mobility, and awareness [27]. In contrast, the donor count was found to be lowest among the oldest age group, which could be explained by the higher prevalence of chronic diseases and higher rates of deferral among older persons [27,28].

The majority of the participants in this study were male donors (97.4%), which is consistent with prior research conducted in Ethiopia [23], Tanzania [25], and India [29,30]. The lower percentage of female donors can be attributed to variables such as menstruation, excessive menstrual bleeding, iron deficiency anemia, and blood loss associated with pregnancy [31-33]. Moreover, differences in blood donation rates between genders may be influenced by societal norms and behaviors [34,35].

The prevalence of blood group O was 44.5%, followed by group B at 26.4%, A at 23.4%, and AB at 5.7%. This distribution of blood groups in Saudi Arabia and other populations is in line with earlier studies, which indicated that O is usually the most prevalent phenotype [16,36-38]. However, there have been documented differences in the proportions of the different ABO phenotypes.

The prevalence of the O blood group in the Hail region aligns with previous research conducted in Saudi Arabia [39] as well as other countries, such as Bahrain [38], India [40], and Bangladesh [41]. Nevertheless, our research outcomes differ from studies conducted in Turkey [42], Palestine [43], Switzerland [44], and Jordan [45], where A was found to be the predominant blood group. Differences in blood group frequencies can mostly be explained by the genetic compositions of populations in specific geographic areas. Our findings on the prevalence of the Rh(D) negative blood type are consistent with those of a similar study conducted in Egypt, another Arabian country, suggesting that this blood type may be evenly distributed throughout the region (11.8% versus 14.4%) [46].

This study used a limited sample size, which may restrict the generalization of the findings on blood type frequency distribution in the studied area. Furthermore, the study did not consider the subtypes of the ABO blood group system. Other limitations include the exclusion of environmental factors, such as the altitude of the Hail region, and genetic variations. Future research should strive to obtain a bigger and more evenly distributed sample by gender.

## Conclusions

According to the results, out of all the ABO phenotypes, O is the most common. B, A, and AB come next. Our findings revealed that Rh(D) positive blood types were significantly more common than Rh(D) negative blood types. This conclusion is consistent with research undertaken in many regions throughout the world, which has shown that the Rh(D) positive phenotype is more prevalent. These results may have considerable consequences for blood transfusion strategies in the Hail region.
